# Intrinsic Dimension Estimation-Based Feature Selection and Multinomial Logistic Regression for Classification of Bearing Faults Using Compressively Sampled Vibration Signals

**DOI:** 10.3390/e24040511

**Published:** 2022-04-05

**Authors:** Hosameldin O. A. Ahmed, Asoke K. Nandi

**Affiliations:** 1 Department of Mechanical and Aerospace Engineering, Brunel University London, London UB8 3PH, UK; hosameldin.ahmed3@brunel.ac.uk; 2 Department of Electronic and Electrical Engineering, Brunel University London, London UB8 3PH, UK; 3 School of Mechanical Engineering, Xi’an Jiaotong University, Xi’an 710049, China

**Keywords:** vibration-based condition monitoring, rolling bearing fault diagnosis, compressive sampling (CS), feature selection, multinomial logistic regression (MLR)

## Abstract

As failures of rolling bearings lead to major failures in rotating machines, recent vibration-based rolling bearing fault diagnosis techniques are focused on obtaining useful fault features from the huge collection of raw data. However, too many features reduce the classification accuracy and increase the computation time. This paper proposes an effective feature selection technique based on intrinsic dimension estimation of compressively sampled vibration signals. First, compressive sampling (CS) is used to get compressed measurements from the collected raw vibration signals. Then, a global dimension estimator, the geodesic minimal spanning tree (GMST), is employed to compute the minimal number of features needed to represent efficiently the compressively sampled signals. Finally, a feature selection process, combining the stochastic proximity embedding (SPE) and the neighbourhood component analysis (NCA), is used to select fewer features for bearing fault diagnosis. With regression analysis-based predictive modelling technique and the multinomial logistic regression (MLR) classifier, the selected features are assessed in two case studies of rolling bearings vibration signals under different working loads. The experimental results demonstrate that the proposed method can successfully select fewer features, with which the MLR-based trained model achieves high classification accuracy and significantly reduced computation times compared to published research.

## 1. Introduction

**Figure 1:**
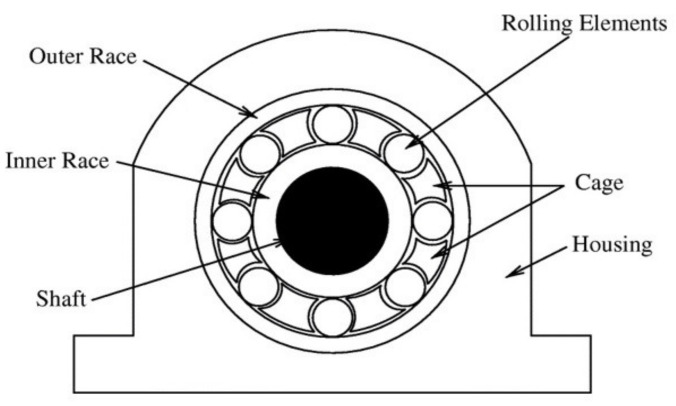
A typical roller bearing [4].

**Figure 2:**
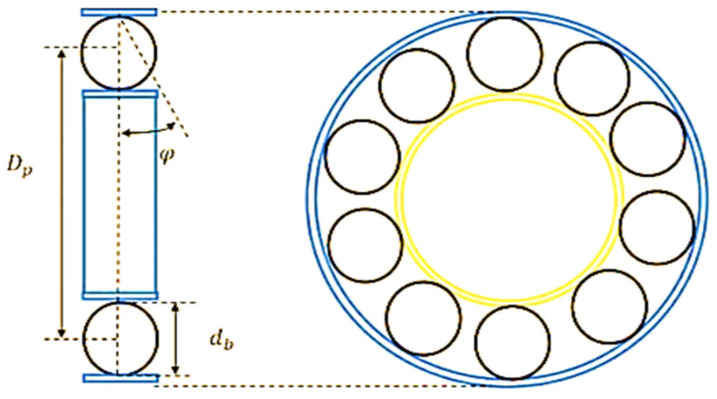
Rolling element bearing geometry [6].

Rolling bearings are critical components of the entire system of rotating machines and play a crucial role in retaining motion between motionless and moving parts. Failure of rolling bearing is one of the key problems in rotating machines that may lead to major catastrophes in machines [1]. It has previously been observed that approximately 40–90% of rotating machines failures are related to bearing faults [2]. Therefore, in most manufacturing procedures, rolling bearings need to be monitored to avoid machine failures. Numerous techniques can be used for machine condition monitoring such as vibration monitoring, electric motor current monitoring, acoustic emission monitoring, etc. Of these, vibration-based condition monitoring has been extensively utilized and has become a widely approved method [3]. As presented in Figure 1, a roller bearing is comprised of some components containing the inner race, the outer race, the rolling elements, and the cage [4]. Bearings faults can happen for several reasons such as fatigue, incorrect lubrication, contamination, corrosion, etc. [5]. In practice, faults in rolling bearings produce a series of impulses that repeat periodically at a rate named the bearing fundamental defect frequency (BFDF), which usually depends on the site of the faults, the geometry of the bearing, and the shaft speed, as displayed in Figure 2 [6].

Based on the damaged component, the BFDFs are categorized into four groups: (i) bearing pass frequency of the inner race (BPFI), (ii) bearing pass frequency of the outer race (BPFO), (iii) ball spin frequency (BSF), and (iv) fundamental train frequency (FTF), which are connected to the defect at the outer race, the inner race, the rolling element, and the cage [7]. These frequencies could be described using the following equations:

(1) $$\mathrm{BPFI}=\frac{N_{b}S_{sh}}{2}\left(1+\frac{d_{b}}{D_{p}}cos\varphi \right)$$

(2) $$\mathrm{BPFO}=\frac{N_{b}S_{sh}}{2}\left(1-\frac{d_{b}}{D_{p}}cos\varphi \right)$$

(3) $$\mathrm{BSF}=\frac{D_{p}}{2d_{b}}\left(1-{\left(\frac{d_{b}}{D_{p}}cos\varphi \right)}^{2}\right)$$

(4) $$\mathrm{FTF}=\frac{S_{sh}}{2}\left(1-\frac{d_{b}}{D_{p}}cos\varphi \right)$$

Here, $N_{b}$ represents the number of rolling elements, $S_{sh}$ represents the shaft speed, $d_{b}$ is the rolling element diameter, $D_{p}$ is the pitch diameter, and φ represents the angle of the load from the radial plane. It has previously been observed that the frequency of the obtained vibration signal specifies the cause of the fault, and the amplitude shows the fault severity.

In vibration-based machine fault diagnosis practice, we handle a huge gathering of vibration signals obtained from several sources in the machines and some background noises. Consequently, it is challenging to use the raw vibration signals directly for fault diagnosis. Much of the current literature on vibration-based fault diagnosis pays particular attention to introducing methods capable of obtaining useful information, usually called features, from the raw vibration signals, which can be successfully used to classify the health condition of the machine. Automatic vibration-based condition monitoring employs machine learning classifiers to classify the vibration signal with its correct health condition type using the obtained features as inputs. Various techniques have been introduced for vibration signal analysis that can be utilized to obtain useful features from the raw vibration data. These include time-domain methods, frequency-domain methods, and time-frequency domain methods.

Numerous studies have examined many statistical techniques and some other cutting-edge methods to extract features from vibration signals in the time domain. For instance, McCormick and Nandi conducted several investigations to classify the condition of a small rotating machine using many statistical parameters such as mean and variance estimated from the time series of vibration signals, which are then applied as inputs to multi-layer perceptron and radial basis function neural networks [8]. Jack and Nandi introduced a genetic algorithm (GA) to select the most significant input features to artificial neural networks (ANN) from a large group of statistical estimates in machine condition monitoring situations [9]. In the same vein, Jack and Nandi attempted to improve the overall generalisation performance of support vector machines (SVMs) and ANNs techniques in two-class fault/no-fault recognition by applying a GA-based feature selection process [10]. In [11] a neural network technique for automated fault diagnosis of rolling element bearing using vibration data was proposed. First, the authors examined ten time-domain features including peak value (Pv), root mean square (RMS), standard deviation (SD), Kurtosis value (Kv), Crest Factor (CrF), Clearance Factor (ClF), Impulse Factor (IF), Shape Factor (ShF), Weibull negative log-likelihood (Wnl), and normal negative likelihood as inputs to ANN. Then only Kurtosis and normal negative likelihood are used as input features to ANN. The results showed that the ANN with Kurtosis and negative normal likelihood performed fault diagnosis with the same accuracy as the ANN with the ten-time domain features that were examined first. Furthermore, Prieto et al. presented a technique for bearing fault detection using statistical time-domain features and ANN. In this technique, several time-domain-based features including, mean, maximum value, RMS, SD, variance, ShF, CrF, latitude Factor, IF, Kv, and normalized fifth and sixth moments were computed from the acquired vibration signals [12].

Previous research has established that frequency domain methods can divulge information based on frequency characteristics that are not certain to be observed in the time domain. Various frequency domain methods have been extensively used for vibration signals analysis in the context of bearing fault classification. For example, McCormick and Nandi examined the application of ANN for rotating shaft’s fault diagnosis using moments of vibration time series as input features. These features were compared with features computed using the fast Fourier transform (FFT) as a suitable choice for real-time implementation [13]. Li et al. introduced a method for motor-bearing fault detection using frequency domain vibration signals and ANN [14]. In this method, the acquired vibration signals in the time domain were converted into the frequency domain using the FFT method. Then, the converted vibration signals in the frequency domain are used as inputs to train the ANN. Zeng and Wang proposed a framework for machine fault classification that comprises data acquisition, data processing, feature extraction, fault clustering, and fault assignment [15]. In this framework, the acquired vibration was transformed into the frequency domain using the FFT. Dhamande and Chaudhari proposed a method for bearing fault diagnosis based on statistical feature extraction in the time and frequency domain and ANN [16]. In this method, many statistical parameters of the vibration data were computed in the time domain including mean, SD, variance, RMS, the absolute maximum of the vibration signal, skewness, kurtosis, CrF, and a combination of them. Additionally, in the frequency domain, several statistical parameters were estimated including the mean, the variance, the third moment, the fourth moment, the grand mean, SD concerning the grand moment, as well as the third and fourth moments concerning the grand mean [16]. Similarly, Helmi and Forouzantabar proposed a technique for rolling bearing fault detection of electric motor applying time domain and frequency domain features with the adaptive neuro-fuzzy interface system (ANFIS) network [17]. In this technique, 15 time-domain features such as mean SD, IF, and skewness were computed. Then, the frequency spectrums were obtained using FFT, and 13 frequency-domain features such as mean, frequency centre, and kurtosis were computed [17].

Moreover, data from several studies suggest that the time-frequency domain methods have been introduced to deal with nonstationary waveform signals, which are very common when machine failures happen. The literature on machines fault diagnosis has highlighted several time frequency methods that are employed to transform the vibration signals in the time domain into the time-frequency domain. For instance, Wang and Chen investigated the sensitivity of three time-frequency domain methods, namely, short-time Fourier transform (STFT), wavelet analysis (WA), and pseudo Wigner–Ville distribution (PWVD) for a rotating machine’s fault diagnosis [18]. In [19], the authors presented a feature extraction methodology that is based on empirical mode decomposition (EMD) energy entropy for rolling element bearings fault diagnosis. In this methodology, a mathematical analysis process to select the most significant intrinsic mode functions (IMFs) was introduced. Then, the selected features were applied as inputs to train an ANN-based model, which is used to classify bearings faults. Furthermore, Djebala et al. presented a denoising technique based on discrete wavelet analysis of the acquired vibration signals for bearing fault detection [20]. In [21], a deep learning-based approach for bearing fault diagnosis is proposed. In this approach, the acquired signals were preprocessed using STFT to generate a spectrum matrix. Then sub-patterns were generated from the spectrum matrix and used to obtain the optimized deep learning structure, the large memory storage retrieval (LAMSTAR) neural network for bearing fault diagnosis. Furthermore, Immovilli et al. introduced a technique for the detection of generalized-roughness bearing fault using the spectral-kurtosis energy of vibration or current signals [22]. Lei and colleagues presented an improved kurtogram method for fault diagnosis of rolling element bearings. In this method, the wavelet packet transform (WPT) was used as the filter of the kurtogram method to overcome the limitations of the original kurtogram [23]. Recently, Hongwei et al. proposed a method for rolling element bearing fault diagnosis based on Fuzzy C-means (FCM) clustering of vibration images that were obtained using EMD-PWVD [24]. In this method, first, the acquired vibration signals with different fault degrees were converted into contour time-frequency images by using the EMD-PWVD technique. Then, the obtained vibration images were divided into sections and their energy distributions values were used as image features. Furthermore, in [25], the authors presented a method for rotating machinery fault diagnosis using time frequency domain features and CNN knowledge transfer.

On the other hand, despite the various techniques described above, which are used to process and examine vibration signals in the time domain, the frequency domain, and the time frequency domain, various studies have proposed other methods that can reduce the computational complexity and enhance fault classification accuracy. Of these, numerous methods have been introduced for learning subspace features from the raw vibration signals in rotating machine fault diagnosis. For instance, in [26], a method is proposed for incipient failures in large-size low-speed rolling bearings using the multiscale principal component analysis (MSPCA) and the ensemble empirical mode decomposition (EEMD). Guo et al. introduced a feature extraction approach for rolling element bearing fault diagnosis using the envelope extraction and the independent component analysis (ICA) technique [27]. In [28], the authors evaluated the use of the principal component analysis (PCA) technique and NN performance for bearing fault diagnosis. In their experiments, the vibration signals were preprocessed using detrended-fluctuation analysis (DFA) and rescaled-range analysis (RSA) techniques. Additionally, Dong et al. introduced a technique for bearing fault diagnosis using kernel PCA (KPCA) and an optimized k-nearest neighbour model [29]. In this technique, first, the original vibration signals were decomposed using local mean decomposition (LMD). Then, the entropy values of product functions that represent the input features were computed utilizing the Shannon method. The KPCA was used to reduce the dimension of the original features needed to train the k-nearest neighbour model [29].

Additionally, data from several studies demonstrate that the use of feature selection techniques will reduce the computational cost and might remove irrelevant and redundant features and accordingly may improve learning performance [1]. Feature selection techniques can be grouped into filter models (e.g., Fisher score, Relief and Relief-F algorithms), wrapped models (e.g., Genetic algorithm), and embedded models (e.g., LASSO and elastic net). Numerous studies have investigated the application of feature selection methods in the context of vibration-based bearings fault diagnosis. For example, Haroun et al. introduced a feature selection method for bearing fault detection and diagnosis using a self-organizing map (SOM) [30]. In this method, the authors, employed multiple methods from the time domain, frequency domain, and time-frequency domains to extract features. Then, Relief-F and minimum redundancy maximum relevance (mRMR) were used to select the optimal features from the extracted features. With these selected features, the SOM was applied to classify the bearing’s health condition [30]. Furthermore, a method for machinery fault diagnosis using redefined dimensionless indicators (RDIs) and mRMR was introduced [31]. In this method, first, the original vibration signals were preprocessed using variation mode decomposition to construct multiple RDIs. Then, the mRMR technique was employed to select several important RDIs. Finally, with these selected RDIs, a grid support vector machine (SVM) was used to carry out the identification of machinery faults. In [32], the authors proposed a methodology for bearing fault diagnosis of induction motors using a genetic algorithm (GA) and machine learning classifiers. In this methodology, first, some statistical features were obtained from the raw signals. Then, the GA was employed to select the most important features. Finally, with the selected features, three different classification algorithms namely, k-nearest neighbour (KNN), decision tree (DT), and random forest (RF) were trained to accomplish the classification task.

Furthermore, recent advances in dimensionality reduction methods have aided the investigation of the compressive sampling (CS) technique [33]. Several researchers have used CS to reduce the dimensionality of the original vibration signals for rolling bearing fault classification. For instance, Wong et al. examined the effects of CS on bearing fault classification [34]. In this investigation, the authors resampled the originally collected vibration signals using a random Bernoulli matrix to match the compressive sampling process. Then, sample entropy-based features were obtained from both the normalized raw vibration signals and the reconstructed signals. Finally, the SVM was trained using the obtained features to accomplish the fault classification task [34]. Tang et al. proposed a CS framework of characteristics harmonics to detect bearing faults [34]. In this method, the characteristics harmonics were obtained from sparse measurements via a compressive matching pursuit technique during the procedure of incomplete reconstruction [35]. Moreover, Xinpeng et al. introduced a bearing fault detection technique using CS and matching pursuit (MP) reconstruction algorithm [36]. In [4], a method for bearing fault classification from highly compressed measurements is proposed. In this method, CS was used to produce highly compressed measurements of the original bearing vibration signals. Then, a deep neural network (DNN) based on sparse autoencoder (SAE) was utilized to learn overcomplete sparse representations of the compressed measurements, which were used for the classification of bearing fault using the Softmax layer. Moreover, Ahmed and Nandi proposed a three-stage method for rolling bearings fault diagnosis using CS and subspace learning techniques [37]. In this method, the CS technique was employed to obtain compressively sampled vibration signals from the original vibration signals. Then, a multistep feature learning algorithm using PCA, linear discriminant analysis (LDA), and canonical correlation analysis (CCA) was used to obtain fewer features from the compressively sampled signals [37]. In [38], a framework for bearing fault classification using CS and feature ranking is proposed. Then, the authors used the CS process to produce compressively sampled signals from the raw vibration signals using two compressible representations of vibration signals, namely, Fourier transform-based coefficients and threshold Wavelet transform-based coefficients. Then, various feature ranking procedures were used to select fewer features from the compressively sampled signals. Finally, three classifiers were evaluated for the classification of bearing faults using these selected features.

The issue of collecting a large amount of vibration data for machine fault diagnosis has attracted considerable attention as it requires large storage to be stored and time to be processed. Existing research has highlighted various techniques for vibration signal analysis that can be applied to obtain useful features from the originally collected vibration data. However, the number of obtained features could be a contributing factor to the performance of these techniques in terms of classification accuracy and computational time, which are particularly important in the real implementation of fault diagnosis techniques. This study aims to contribute to this growing area of research by investigating the following: 1. A method that can reduce the high dimensionality of the raw vibration data to a fewer number of features capable of achieving high fault classification accuracy and highly reduced computational time; 2. The CS is an appropriate mechanism to compress the original high dimensional vibration data and then further reduce the dimension of the compressed vibration data to a far fewer number of features that satisfactorily represent the health condition of rolling bearings, and 3. The multinomial logistic regression (MLR) algorithm is a possible classifier to deal with the bearing’s fault classification task using the fewer selected features.

To accomplish high classification accuracy and highly reduced computation time, this paper proposes a new methodology for bearing fault classification based on intrinsic dimension estimation-based feature selection and multinomial logistic regression using compressively sampled vibration signals. In the methodology, the input vibration signals are resampled using CS to reduce the high-dimensional samples of the originally collected vibration data. Then, to further reduce the number of features of the compressively sampled vibration signals to far fewer features that can sufficiently represent the health condition of rolling bearings consequently achieve high classification accuracy and highly reduced computation time, a feature selection procedure based on intrinsic dimension estimation, stochastic proximity embedding (SPE), and neighbourhood component analysis (NCA) is applied. Finally, to perform the bearing’s fault classification task, the fewer selected features are applied as inputs to a multinomial logistic regression (MLR) classification algorithm. The contributions of this paper are as follows:

1. The proposed method produces far fewer features that can represent the health condition of bearings. This study accomplishes high classification accuracies and highly reduced computational time with regression analysis-based predictive modelling technique, namely the multinomial logistic regression (MLR) classifier using the fewer selected features as inputs.
2. A dimensionality reduction process has been proposed, which comprises (1) data compression using CS and (2) intrinsic dimension estimation-based feature selection process, which includes (a) SPE-based feature selection that utilizes a self-organizing iterative scheme to embed the compressed data dimension into a further lower dimension, and (b) the non-parametric NCA-based feature selection that maximizes the stochastic variant of the leave-one-out nearest neighbour score to achieve the best classification accuracy on the training set. This ensures selecting fewer features from the high dimensional data capable to achieve high classification accuracy and a reduced computational time.
3. We studied the impact of values of two parameters within the data compression and feature selection process used in the proposed method, namely the compressive sampling rates and the NCA tolerance values on the number of the selected features and the fault classification accuracy.
4. Two fault classification case studies of rolling element bearings vibration signals under different working loads are used to evaluate the proposed method.
5. Compared to recently published classification results from the literature on the same vibration-bearing datasets used in this study, our proposed method achieves high classification accuracy and highly reduced computation time, which suggests that our proposed methodology could be used in actual applications of vibration-based machine fault diagnosis.

The remainder of this paper is organized as follows. Section 2 describes the proposed framework. Section 3 is devoted to descriptions of the experimental study used to validate the proposed framework and presents comparison results. Finally, Section 4 offers some conclusions.

## 2. The Proposed Method

**Figure 3:**
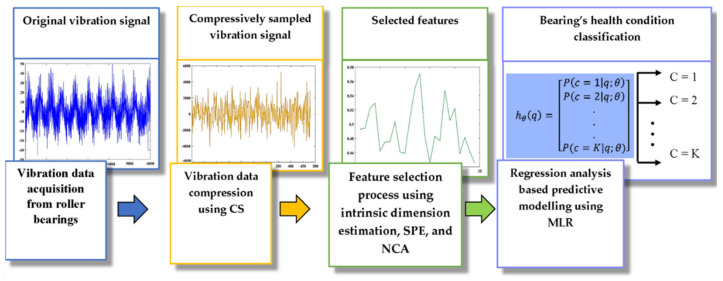
The proposed method.

The methodological approach taken in this study is a mixed methodology based on vibration data compression, feature selection based on intrinsic dimension estimation, and regression analysis-based predictive modelling techniques. The proposed method automatically learns and selects far fewer features from compressively sampled vibration signals, which can be used as inputs to a classifier for bearing fault detection and classification. The key objective of the proposed method is to achieve high fault classification accuracy while highly reducing the computation time. The flow chart of the proposed method is presented in Figure 3. First, the compressive sampling (CS) mechanism was employed to compress the acquired vibration data. Then, a feature selection procedure based on intrinsic dimension estimation, stochastic proximity embedding (SPE), and neighbourhood component analysis (NCA) was utilized to estimate and further reduce the dimensionality of the compressively sampled data. Finally, with these reduced features, a classifier was used to classify the bearing’s health condition, namely, the multinomial logistic regression (MLR) algorithm was employed to perform the classification task. The following subsections discuss the proposed method in more detail.

### 2.1. Vibration Data Compression Using CS

To reduce the high dimensional of the collected vibration data, the proposed method uses the CS mechanism to obtain compressively sampled vibration signals from the original signals. The central principle of the CS is that a finite-dimensional signal having sparse or compressible representations can be reconstructed from a small number of linear measurements much lower than measurements based on the Nyquist sampling rate {xe “Nyquist sampling rate”}. Machine vibration signal has compressible representations in several domains such as in the frequency domain using FFT {xe “frequency domain”}. Therefore, in recent times, there has been a growing interest in the application of CS in machine fault diagnosis {xe “*fault diagnosis*”}. There are many benefits of CS in vibration-based bearing fault diagnosis, e.g., reducing the high dimension of the acquired vibration data, reducing computation time required to analyze the collected data, reducing data transmission cost in the cases where it is essential to send the collected data from remote places, e.g., fault diagnosis of offshore wind turbines.

The successful implementation of the CS mechanism is based on two fundamental concepts: (i) the sparsity of the targeted signal, and (ii) the measurements matrix that fulfils the minimal data information loss, which is usually called the restricted isometry property {xe “restricted isometry property”} (RIP) [39]. Briefly, we describe the CS mechanism as follows.

Let $x\in \;R^{n\;x\;1}$ be the originally collected time-indexed signal. With an identified sparsifying transform {xe “sparsifying transform”} matrix $\psi \;\epsilon \;R^{n\;x\;n}$ where the columns represent the basis elements ${\left\{\psi_{i}\right\}}_{i=1}^{n}$, the signal *x* can be described as follows,

(5) $$x=\overset{n}{\underset{i=1}{\sum }}\psi_{i}s_{i}$$

or,

(6) x=ψs

Here, s represents a n∗1 column vector of coefficients. In case the basis ψ generates q-sparse representations of the signal x then x of length n can be signified with q<<n nonzero coefficients. Consequently, Equation (5) can be rewritten as follows,

(7) $$x=\overset{q}{\underset{i=1}{\sum }}\psi_{ni}s_{ni}$$

Here, ni represents the index of the basic elements and the coefficients corresponding to the q nonzero elements. Accordingly, $s\;\epsilon \;R^{n\;x\;1}$ represents a vector column with q nonzero elements and characterizes the sparse representation {xe “sparse representation”} vector of the signal x. Consistent with the CS mechanism, with m<<n projections of the vector x, measurement vectors ${\left\{\emptyset_{j}\right\}}_{j=1}^{m}$, and the sparse representations s, the compressed measurements of the signal x can be obtained using the following equation,

(8) y=∅ψs=θs

**Figure 4:**
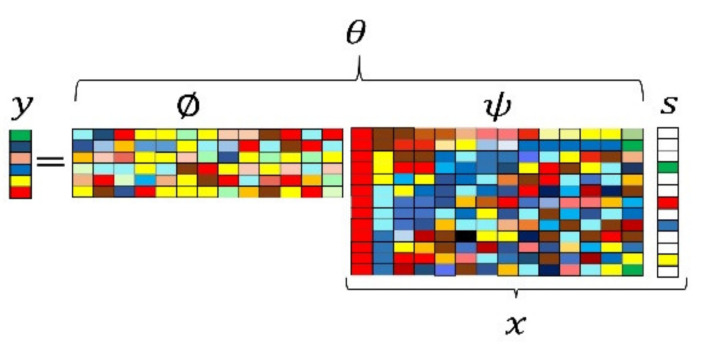
Single measurement vector compressive sampling framework [37].

Here, y is a m∗1 column vector of the compressed measurements and θ=∅ψ represents the measurement matrix. Figure 4 shows an illustration of the CS framework that can be used to produce the single measurement vector of the compressed measurements y. According to the CS theory, the original signal x can be reconstructed from the compressed measurements y by applying a recovery algorithm. This can be completed by first recovering the sparse representation vector s and then employing the inverse of the sparsifying transform {xe “sparsifying transform”} ψ to recover x. One of the solutions to be used to recover the sparse representations $s\in R^{n}$ from its compressed measurement vector $y\in R^{m}$ is the $l_{0}$ minimization technique, which searches for a sparse vector consistent with the measured data y=θs such that,

(9) $$\hat{s}=\underset{z}{\arg\;\min}{\left\|s\right\|}_{0}\;\mathrm{such}\;\mathrm{that}\;\theta s=y$$

Moreover, the convex optimization ${\left\|.\right\|}_{1}$ can also be employed in place of ${\left\|.\right\|}_{0}$, such that,

(10) $$\hat{s}=\underset{z}{\arg\;\min}s_{1}\;\mathrm{such}\;\mathrm{that}\;\theta s=y$$

In case the measurement matrix θ satisfies the {xe “Restricted Isometry Property”} RIP, the sparse representation s can be reconstructed by solving the convex program in Equation (10). The matrix θ satisfies the *r*th restricted isometry property {xe “Restricted Isometry Property”} (RIP) if there exists a $\delta_{r}\ll 1$, such that

(11) $$\left(1-\delta_{r}\right){\left\|s\right\|}_{l_{2}}^{2}\leq {\;\|\theta s\|}_{l_{2}}^{2}\leq \left(1+\delta_{r}\right){\;\|s\|}_{l_{2}}^{2}$$

In our case, the collected vibration data is a collection of signals, which can be represented as a matrix of sparse vectors Y, such that,

(12) Y=ӨS

where $Y\in R^{m\;x\;L}$, L is the number of observations and m is the number of compressed measurements, $Ө\in R^{m\;x\;n}$ represents a dictionary, and $S\;\in R^{n\;x\;L}$ is a sparse representation {xe “sparse representation”} matrix. Therefore, multiple measurement vector compressive sampling {xe “Compressive Sampling”} is used in our proposed method.

Our proposed method is intended to learn directly from the compressed vibration signals. To obtain the compressively sampled signals from the collected vibration dataset $X=\left\{x_{1},x_{2},\;\ldots ,\;x_{L}\right\}$ $\in R^{n\;}$, first, the {xe “Fast Fourier Transform”} FFT, which commonly provides a sparse basis for vibration signals, is employed to produce the sparse representation ($S\in R^{nxL}$) that comprises only a small number q ≪n  of nonzero coefficients. The FFT algorithm calculates the n-point complex discrete Fourier transform (DFT) of the signal X. In this study, we utilise the magnitude of the DFT to get *S*. Then, a random matrix with i.i.d Gaussian entries matrix, which satisfies the RIP, is used as the measurement matrix $\;Ө\in R^{mxn}$ [40]. Finally, a compressed sampling rate (*α*) is used to produce the compressively sampled signals $Y\in R^{mxL}$, where *m* represents the number of compressed signal elements and given by m=α∗n. This compression process is summarized in Algorithm 1 below:**Algorithm 1** Vibration data compression using CS
1. Input: vibration dataset
$X\in R^{n\;x\;L};$ measurement matrix
$Ө\in R^{mxn}$ and compressive sampling rate
α

2. Output: compressively sampled vibration signals
$Y\in R^{m\;x\;L}$
3. Produce the sparse representations S of *X*: $abs\;\left(FFT\left(X\right)\right)$$\;\to \;S\in R^{n\;x\;L}$
4. Project
S into Ө with compressed sampling rate
α to obtain compressively sampled to obtain compressively sampled signals
$Y\in R^{m\;x\;L}$


### 2.2. Feature Selection Process

Based on CS theory, the compressively sampled signals (Y) have sufficient information to reconstruct successfully the originally collected vibration signals. Nevertheless, the dimensions of these compressively sampled vibration signals might be further reduced to attain more reduction in the computational cost while achieving high classification accuracy. Accordingly, our proposed method offers a feature selection process to learn and select fewer features from the compressively sampled signals (Y) to achieve superior classification accuracy and reduced computation costs. The feature selection process starts by identifying the minimal number of features required to represent the compressively sampled vibration signals Y, using a global dimension estimator, namely the geodesic minimal spanning tree (GMST). The GMST calculates the geodesic graph *G* from which the intrinsic dimension (*d*) is projected by calculating multiple minima spanning trees (MSTs) in which each data sample $x_{i}$ is linked to its *k* nearest neighbours [41], such that,

(13) $$d\left(Y\right)=min\underset{e\in T}{\sum }D_{Eucl}$$

Here, *T* signifies the set of all the subtrees of *G*, *e* is an edge in *T*, and $D_{Eucl}$ is the Euclidean distance of *e*. Then, with the computed minimal number of features *d,* where *d* < *m*, the SPE technique is employed to convert the compressively sampled data $\left(Y\right)$ into a reduced-dimensionality space of significant representation $\left(Z\;\in R^{d\;x\;L}\right)$.

The SPE is a nonlinear approach that has many benefits such as being simple to implement, very fast, scales linearly with the size of the data in both time and memory, and is relatively insensitive to missing data [42]. Thus, it was decided that SPE is an appropriate method to use for this investigation. The SPE utilizes a self-organizing iterative scheme to embed *m*-dimensional data into *d* dimensions, such that the geodesic distances in the original *m* dimensions are preserved in the embedded *d* dimension. Briefly, we describe the simplified SPE procedure as follows [43]:

1. Initialize the coordinates $y_{i}$. Select an initial learning rate β.
2. Select a pair of points, i, and j, at random, and calculate their distance: $\;d_{ij}=\|y_{i}-y_{j}\|$. If $\;d_{ij}\;\neq r_{ij}$ ($r_{ij}$ is the distance of the corresponding proximity), update the coordinates $y_{i}$ and $y_{j}$ using the following equations,
  (14) $$\;y_{i}\leftarrow \;y_{i}+\beta \frac{1}{2}\;\frac{r_{ij}-d_{ij}}{\;d_{ij}+\upsilon }\;\left(y_{i}-y_{j}\right)$$
  and
  (15) $$y_{j}\leftarrow \;y_{j}+\beta \frac{1}{2}\;\frac{r_{ij}-d_{ij}}{\;d_{ij}+\upsilon }\;\left(y_{j}-y_{i}\right)$$

Here, υ is a small number to avoid division by zero. For a given number of iterations, this step will be repeated for several steps and β will be reduced by a recommended decrement δβ. Finally, to obtain far fewer selected features in the feature selection process step, our proposed method uses the NCA technique to automatically selects a subset from the SPE-based learned features by converting $Z\;\in R^{d\;x\;L}$ into *Q* $\in R^{f\;x\;L}$ where f<d. Briefly, we describe the NCA feature selection as follows:

Let $Z=\left\{\left(z_{1},\;c_{1}\right),\;\ldots ,\;\left(z_{i},\;c_{i}\right),\;\ldots ,\;(z_{L},\;c_{L}\right\}$ be a training set samples with *d*-dimension and $c_{i}\in \left\{1,\;\ldots ,\;C\right\}$ is the matching class label. The NCA searches for a weighting vector w that uses to select a feature subset. In this method, first, the weighted distance between two samples $z_{i}$ and $z_{j}$ can be computed using the following equation,

(16) $$D_{w}\left(z_{i},\;z_{j}\right)=\overset{d}{\underset{r=1}{\sum }}w_{r}^{2}\left|z_{il}-z_{jl}\right|\;$$

Here, $w_{r}$ is a weight-related to the *r*-th feature. Then, the strategy is to maximize the leave-one-out classification accuracy on the training set. The reference point is defined by a probability distribution. In our case, the probability of $z_{i}$ chooses $z_{j}$ as its reference point such that,

(17) $$p_{ij}=\left\{\begin{array}{c} \frac{k\left(D_{w}\left(z_{i},\;z_{j}\right)\right)}{\sum_{k\neq i}k\left(D_{w}\left(z_{i},\;z_{j}\right)\right)},\;\mathrm{if}\;i\neq j\\ 0,\;\mathrm{if}\;i=j\; \end{array}\right.$$

Here, $k\left(D_{w}\left(z_{i},\;z_{j}\right)\right)=\exp\left(-(D_{w}\left(z_{i},\;z_{j}\right)\right)/\sigma )$ is a kernel function and σ is an input parameter that represents the kernel width. The probability of correct classification of $y_{i}$ can be computed using the following equation,

(18) $$p_{i}=\underset{j}{\sum }c_{ij}p_{ij}\;$$

Here,

(19) $$c_{ij}=\left\{\begin{array}{c} \;1\;\mathrm{if}\;c_{i}=c_{j}\\ \;0\;\mathrm{otherwise} \end{array}\right.$$

This process of NCA is summarized in Algorithm 2 below [44]:
**Algorithm 2** NCA Feature Selection
1. Input: $Z\;\in R^{d\;x\;L};\;\gamma$ initial step length; σ: kernel width; ℷ: regularisation parameter; and *η*: small positive constant. 2. Initialization: $w^{\left[0\right]}=\left(1,\;1,\;\ldots ,\;1\right),\;\epsilon^{\left(0\right)}=-\infty$, t=0. 3. Repeat 4. for i=1, …, L  do 5. Compute $p_{ij}$ and $p_{i}\;$ using
$w^{\left(t\right)}$ according to (2) and (3) 6. for r=1, …, d   do 7. $\nabla \_r=2(1/\sigma (p_{i}\underset{j\neq i}{\sum }p_{ij}\left|z_{ir}-z_{jr}\right|-\underset{j}{\sum }c_{ij}\left|z_{ir}-z_{jr}\right|)-ℷ)w_{r}^{\left(t\right)}$ 8. *t* = *t* + 1 9. $w^{\left(t\right)}=w^{\left(t-1\right)}+\gamma \Delta$ 10. $\epsilon^{\left(t\right)}=\xi \left(w^{\left(t-1\right)}\right)$ 11. $\mathrm{if}\;\epsilon^{\left(t\right)}>\epsilon^{\left(t-1\right)}\;\mathrm{then}\;\gamma$ = 1.01 γ 12. else 13. γ=0.4γ 14. $\mathrm{until}\;\left|\epsilon^{\left(t\right)}-\epsilon^{\left(t-1\right)}\right|<\eta$ 15. $w=w^{t}$ 16. Return w


To select the most important features based on features weights, the criteria are based on a threshold value (*Thr*), which can be computed as follows:

(20) $$Thr=\tau \;\max\left(w\right)\;$$

Here τ is the tolerance value.

### 2.3. Regression Analysis-Based Predictive Modelling Using MLR

Regression analysis can be used as a predictive modelling method as it defines the relationship between a dependent variable and one or more independent descriptive variables. There are many types of regression analysis methods such as linear regression, polynomial regression, logistic regression, etc. Of these, logistic regression (LR) is one of the most used methods in many machine learning applications. The LR is usually utilized for binary classification, i.e., the class labels c has only two values, e.g., (Fault, Normal). Briefly, we describe the LR as follows:

Let a training data $Q=\left\{q_{1},\;q_{2},\;\ldots ,\;q_{L}\right\}$ with f-dimension produced in the feature selection step of our proposed method. The LR is a probabilistic discriminative model that learns P(c|Q) directly from the training data where $c_{i}\in \left\{0,\;1\right\}$, such that,

(21) $$P\left(c=1|q\right)=h_{1}\left(q\right)=g\left(-\theta^{T}h\right)=1/\left(1+e^{-\theta^{T}q}\right)\;$$

Here $g\left(-\theta^{T}h\right)$ is the logistic function, which is also called the sigmoid function. Since $\sum P\left(C\right)=1$, we can compute $P\left(c=0|Q\right)$ as follows:

(22) $$P\left(c=0|q\right)=h_{0}\left(q\right)=1-\;P\left(c=1|q\right)=1-(1/(1+e^{-\theta^{T}q}))$$

The likelihood of the parameters of L training examples can be computed using the following equation,

(23) $$L\left(\theta \right)=\overset{L}{\underset{i=1}{\prod }}({\left(g\left(\theta^{T}q^{i}\right)\right)}^{c\left(i\right)}{\left(1-g\left(\theta^{T}q^{i}\right)\right)}^{1-c\left(i\right)}\;$$

Here $\theta =\left[\theta_{0},\;\theta_{1},\;\ldots ,\;\theta_{i}\right]$ represents the parameters of the model. Nevertheless, the log-likelihood is widely utilised and, consequently, Equation (23) and can be updated as Equation (24), such that

(24) $$\log\;L\left(\theta \right)=\overset{L}{\underset{i=1}{\sum }}\log(({\left(g\left(\theta^{T}q^{i}\right)\right)}^{c\left(i\right)}\left(1-g\left(\theta^{T}q^{i}\right){)}^{1-c\left(i\right)}\right)\;$$

To avoid overfitting a regularisation term, λ is added to the log-likelihood function, such that

(25) $$\log\;L\left(\theta \right)=\overset{L}{\underset{i=1}{\sum }}\log\left(P\left(c^{i}=c_{k}|q^{i};\theta \;\right)\right)-\frac{\lambda }{2}{\left\|\theta \right\|}^{2}$$

The MLR classifier, which also goes with the name SoftMax regression in ANN, generalizes the LR to a multi-class classification problem with multi-labels $c^{\left(i\right)}\;\in \left\{1,\ldots ,K\right\}$, such that

(26) $$\;h_{\theta }\left(q\right)=\left[\begin{array}{c} P\left(c=1|q;\theta \right)\\ P\left(c=2|q;\theta \right)\\ .\\ .\\ .\\ P(c=K|q;\theta ) \end{array}\right]=\frac{1}{\sum_{j=1}^{K}\exp\left(\theta^{\left(j\right)T}q\right)}\;\left[\begin{array}{c} e^{\theta^{\left(1\right)T}q}\\ e^{\theta^{\left(2\right)T}q}\\ .\\ .\\ .\\ e^{\theta^{\left(K\right)T}q} \end{array}\right]$$

Here, $\;\theta^{\left(1\right)}$,$\;\theta^{\left(2\right)},\;\ldots ,\;\theta^{\left(K\right)}\;$ represent the parameters of the multinomial logistic regression model. In this study, we are dealing with a multiclassification problem, so MLR is employed to perform the classification task in our proposed method.

## 3. Experimental Study

Two fault classification case studies of rolling element bearings using vibration signals are presented to evaluate the proposed method.

### 3.1. First Case Study

**Figure 5:**
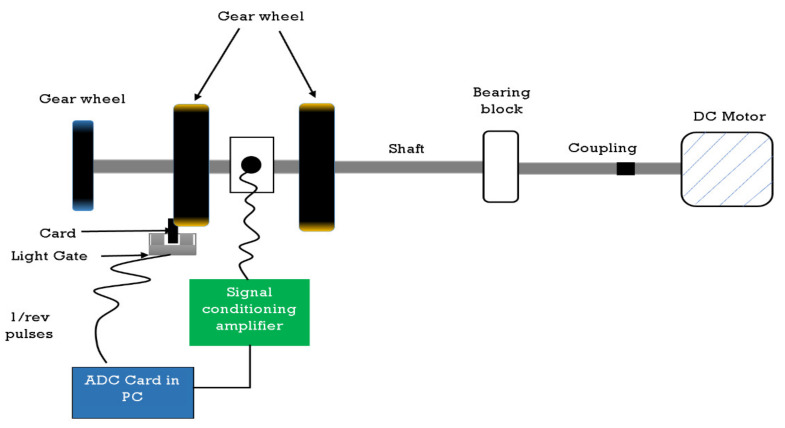
The test rig used to collect the vibration data of bearings of the first case study [4].

The vibration dataset used in this case study is acquired from experiments on a test rig that simulates running roller bearings’ environment. In these experiments, several interchangeable faulty roller bearings are inserted in the test rig to symbolize the type of faults that can normally happen in roller bearings. As shown in Figure 5, the test rig to collect the vibration dataset of bearings contains a 12V DC electric motor driving the shaft via a flexible coupling. The shaft was supported by two blocks of Plummer bearing, where several damaged bearings were inserted. Two accelerometers were used to measure the vibration signals in both the horizontal and vertical planes. The output from the accelerometers was fed back over a charge amplifier to a Loughborough Sound Images DSP32 ADC card with a low-pass filter using a cut-off of 18 kHz. The sampling rate was 48 kHz. Six health conditions of roller bearings have been recorded with two normal conditions {XE “normal conditions”}, i.e., brand new condition (NO) and worn yet undamaged condition (NW), and four faulty conditions {XE “faulty condition”} with, inner race fault {XE “inner race fault”} (IR), an outer race fault {XE “outer race fault”} (OR), rolling element fault {XE “rolling element fault”} (RE), and cage fault (CA). Table 1 explains the corresponding characteristics of these bearing health conditions [4].

**Figure 6:**
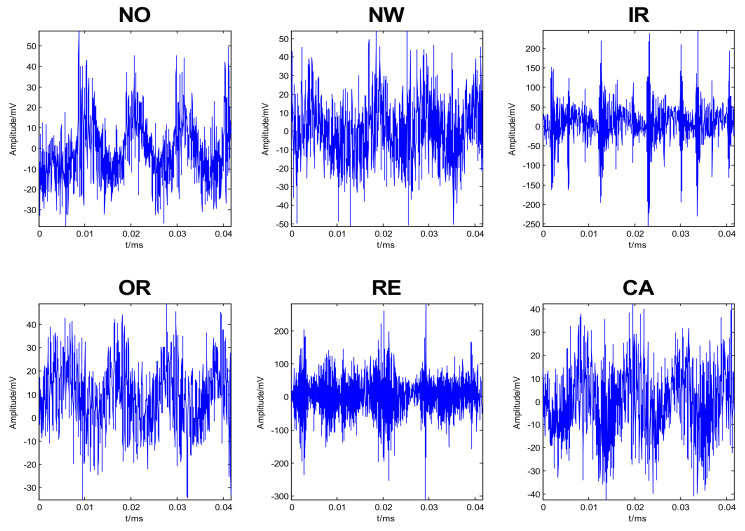
Typical time domain vibration signals for the six different conditions [4].

The data was recorded using 16 different speeds within 25–75 rev/s. In each speed, ten time series were recorded for each condition, i.e., 160 examples per condition. This resulted in a total of 960 examples with 6000 data points each. Figure 6 illustrates some typical time series plots for the six different conditions.

To apply our proposed method in this case study, first, we randomly selected 50% of the total observations for training, and the remaining 50% is employed for testing the trained model. Then, we computed the compressively sampled vibration signal from the high dimensional data X which has 6000 time samples for each of its 960 observations. As described in Algorithm 1, the FFT basis was used as the sparse representation of each signal in X. After, the CS framework with different sampling rates (*α*) (0.1, 0.2, and 0.3) using a random Gaussian matrix was used to produce the compressed measurements.

**Figure 7:**
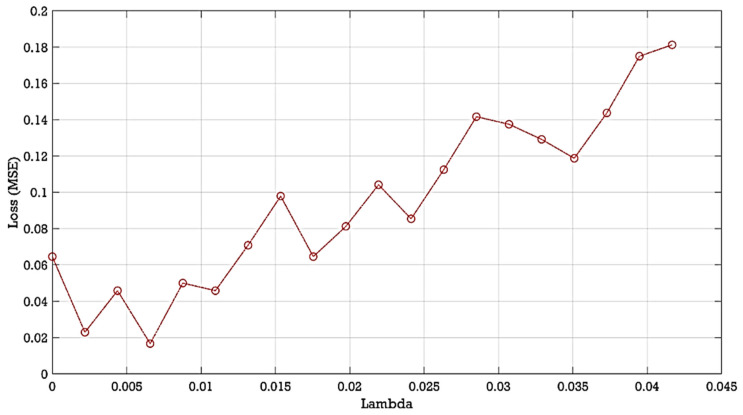
Example of the average loss values versus λ values computed from the reduced dimension of compressively sampled data with *α* = 0.2.

**Figure 8:**
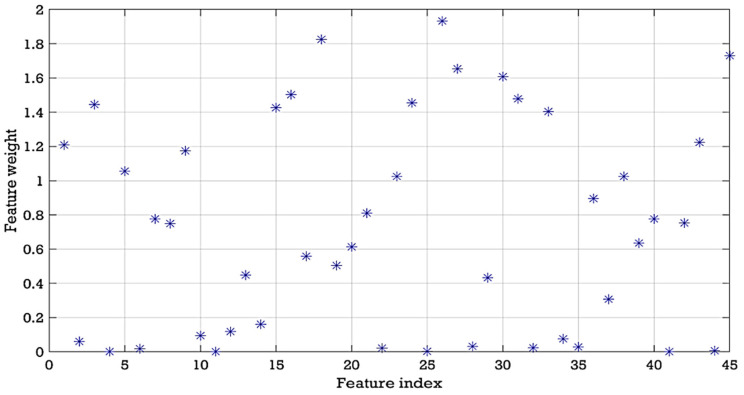
Example of the selected features and their corresponding weights using *α* = 0.2 and NCA tolerance value = 0.02.

To estimate and reduce further the dimensionality of the compressively sampled signals, first, we computed the intrinsic dimension (*d*) using the GMST technique. Then, the process combining the SPE and NCA techniques was performed to learn and select far fewer features from the compressively sampled vibration signals. The compressively sampled vibration signals were transformed into further reduced dimensions using the defined intrinsic dimension and the SPE method. Then, a regularized NCA based on feature weights and a relative threshold was employed to select far fewer features with *f*-dimension from the SPE-based selected features with *d*-dimension where *f < d*. We computed the best regularization parameter $\left(\lambda \right)$ value that corresponds to the minimum average loss to be used in fitting the NCA model on all the reduced dimension data. The final selected features were computed using the feature weights of the NCA model and a relative threshold. The stochastic gradient descent (SGD) solver was used for estimating feature weights. Two tolerance values (0.01 and 0.02) were tested in this case study for computing the threshold values used in the feature selection process. Figure 7 shows an example of the average loss values versus λ values. Figure 8 presents an example of the selected features and their corresponding weights. Moreover, Table 2 shows examples of the computed values of the average intrinsic dimension, the dimension of the NCA-based selected features, least loss, and best λ values taken from 10 trials.

The first benefit of the proposed method is to obtain far fewer features from the acquired vibration signals to be successfully used for rolling bearing fault diagnosis and consequently reduce the computational cost. Therefore, the first set of analyses examined the impact of the tolerance values and the compressive sampling rates on the number of the selected features using our proposed method. As shown in Table 2, the average least losses were slightly reduced from 0.014 to 0.013; 0.013 to 0.009; and from 0.013 to 0.010 using the tolerance value of 0.01 in place of 0.02. Furthermore, with a 0.02 tolerance value, the computed average best lambda value for NCA remained the same for all the compressive sampling rates (with λ=0.003), while for the tolerance value of 0.01, the value λ=0.004 achieved for the sampling rate of α=0.3, and λ=0.004 was obtained for both α=0.1 and α=0.2.

Moreover, as Table 2 shows, all the computed average least lost values are very small—in the range of 0.009–0.014—although the feature dimension was reduced from 6000 (in the original raw vibration signals) to 600 (the dimension of the compressively sampled signals with *α* = 0.1), which reduced to 28 (*d-dimension*) and then further reduced to 8 (*f-dimension wit 0.01 tolerance value*). This suggests that the feature selection is a good idea to be performed in our proposed method.

Furthermore, the NCA tolerance values have a clear effect on the computed intrinsic dimension (*d*) and the dimension of the NCA-based selected features. For example, with a 0.02 tolerance value, we obtained intrinsic dimension (*d*) of 62, 55, 33, which reduced to 28, 40, and 26 with 0.01 tolerance value for α=0.1, α=0.2, and α=0.3, respectively. Furthermore, with the tolerance value of 0.02, we obtained the final feature dimension *(f)* of 18, 14, and 11, which reduced to 8, 10, 8 with 0.01 tolerance for α=0.1, α=0.2, and α=0.3 respectively. Taken together when the tolerance value decreases the dimension of both selected features, i.e., *d* and *f*, decreases.

The final NCA-based selected features were used to train the classification algorithm, i.e., the MLR, to classify among *c* classes of roller bearing health conditions. The overall results are shown in Table 3, where the classification accuracy is the average of 10 trials for each experiment and the time obtained by averaging the training time and testing time of these 10 trials. It is apparent from this Table that our proposed method achieved high classification accuracies (all above 99%) for all the compressive sampling rates and tolerance values with less than 35% of the acquired vibration data samples. Classification accuracies from our proposed method are 99.9%, 99.7%, and 99.5% for only 30%, 20%, and 10% of the whole collected data with 8, 10, 8 selected features (with tolerance value = 0.01) respectively, used to train the MLR classifier. Additionally, with tolerance value = 0.02, α=0.3, and 11 selected features, the proposed method achieved 100% classification accuracy for every single run in our experiments. Moreover, the trained classification model of our proposed method requires less than 0.016 s to complete the classification task.

Table 4 provides sample confusion matrices of the classification results of MLR classifier using selected features with tolerance value = 0.01 and a sampling rate of (a) α=0.1, 0.2, and α=0.3. As can be seen from Table 4c, the recognition of the bearing health conditions with α=0.3 is 100%. In Table 4a, with 10% testing data (with α=0.1), our method misclassified one of the testing examples of condition 5, i.e., RE, as condition three, i.e., IR. In Table 4b, with 20% testing data (with α=0.2), our method misclassified only two of the testing examples of condition 2 (NW) as condition 6 (CA).

#### Comparisons of Results

In this subsection, a comparison of various methods using the same vibration dataset of rolling bearings used in the first case study (see Table 5). In [34], three methods were used for bearing fault diagnosis using SVM. The first method used the whole collected vibration data. The second method used compressively sampled datasets of α=0.25 and α=0.5, while the third method used the corresponding reconstructed signals of these compressively sampled data. In [45], a method using a genetic programming (GP) algorithm for feature extraction was used, and then ANN and SVM were employed to classify bearing health conditions. In [46], a hybrid model comprising the fuzzy min–max (FMM) neural network and random forest (RF) with sample entropy (SampEn) and power spectrum (PS) features was utilized to classify bearing health conditions. In [37], a three-stage hybrid method consisting of CS, PCA, LDA, and canonical correlation analysis (CCA) was used for bearing fault classification from (1) the whole 6000 samples from the frequency domain, and (2) compressively sampled data with α=0.1 and α=0.2. In [38], a framework combining CS and feature ranking techniques including fisher score, Laplacian score, Relief-F, Pearson correlation coefficients, and Chi-square (Chi-2) were used for bearing fault classification from compressively sampled vibration data with 0.1, feature dimension of 120. Then, with these features, the MLR classifier was used to classify bearing faults. In [47], a three-stage method combining CS (with α=0.1, 0.2, and 0.3), a feature selection procedure, and SVM was used for bearing fault classification.

As Table 5 shows, the classification results of our proposed method are better than those reported in [34,45]. Moreover, our results are the same as, if not better than the classification results described in [37,38,46,47]. Our method is extremely fast and needs only 0.015 s to complete the fault classification task compared to the method in [37], which needs 6.7 s using a classification model trained with 10% of the whole data. Furthermore, results from our proposed method remain as good as, if not well improved than the results stated in [38], although we used limited features (only 8 features), which are not met by the method in [38], which used 120 features.

For further verification of the efficacy of the proposed method, we conducted three experiments using our proposed method using the same settings used to perform our experiments with α=0.1, 0.2, and 0.3. Then, we employed SVM in place of the MLR classifier in our proposed method to classify bearing health conditions to examine the speed and accuracy performance of our proposed method compared to the method used in [47]. The results are presented in the last row of Table 5. The classification results of our method with MLR classifier are as good as, if not better than the results of our method with SVM. Interestingly, the results demonstrate that our method with MLR classifier is faster and requires only 25%, 13.3%, and 10% of the time of our method with SVM to complete the classification task using classification models trained with *α* = 0.1, 0.2, and 0.3, respectively.

### 3.2. Second Case Study

The bearing datasets used in this case are provided by Case Western Reserve University (https://engineering.case.edu/bearingdatacenter/download-data-file, accessed on 2 April 2022). The bearing datasets were obtained from a motor driving mechanical system where the faults were planted into the drive-end bearing of the motor under four different speeds and several health conditions, namely, normal condition (NO), with an IR fault (IR), with a roller element fault (RE), and with an OR fault (OR). Then, the datasets were further categorized by the width of the fault (0.18–0.71 mm) and the load of the motor (0–3 hp). The sampling rates {xe “sampling rates”} utilized were 12 kHz for some of the sampled data and 48 kHz for the rest of the sampled data. At each speed, 100 time series were recorded for each condition per load. For the IR, OR, and RE conditions, vibration signals for four different fault widths (0.18, 0.36, 0.53, and 0.71 mm) were separately recorded. In this study, of these acquired vibration signals, two groups of datasets were prepared for the evaluation of our proposed method.

The first group of datasets is selected from the data files of the vibration signals sampled at 48 kHz with fault width (0.18, 0.36, and 0.53 mm) and fixed loads including 1, 2, and 3 hp, and the number of examples chosen is 200 examples per condition. This offered three different datasets A, B, and C with 2000 total examples and 2400 data points for each signal. The second type of bearing dataset was chosen from the data files of vibration signals sampled at 12 kHz with fault size (0.18, 0.36, 0.53, and 0.71 mm) and load 2 hp, and the number of examples chosen was 60 examples per condition. This offered a dataset D with 720 total examples and 2000 data points for each signal. The description of the used bearing vibration dataset is presented in Table 6.

To classify the bearing’s health conditions from datasets A, B, C, and D described above, the same steps as in the first case study were followed to apply our proposed method. First, 50% of the total observations of datasets A, B, C, and D were randomly chosen for training and the other 50% is employed for testing the trained model. Then, we obtained the compressively sampled vibration signal from the high dimensional datasets A, B, and C, with 2400 time samples for each of the 2000 observations and dataset D with 2000 time samples for each of the 720 observations. The FFT coefficients were employed as sparse representations of all the datasets used in the second case study, i.e., A, B, C, and D. Then, we adopted the CS mechanism with different sampling rates (*α*) of 0.1, 0.2, and 0.3, using a random Gaussian matrix as described in the first case study to obtain the compressively sampled signals for each dataset.

To estimate and reduce further the dimensionality of the compressively sampled signals, we applied the same steps of the feature selection process step of our proposed method as described in the first case study. Two tolerance values (0.01 and 0.02) were investigated for the feature selection process. Table 7 presents examples of the computed values of the average intrinsic dimension, the dimension of the NCA-based selected features, least loss, and best λ values taken from 10 for each dataset. To test the impact of the tolerance values and the compressive sampling rates on the number of the selected features using our proposed method. Table 7 shows the computed values of the average intrinsic dimension (*d*), the dimension of the NCA-based selected features (*f*), the best least loss, and best λ values taken from 10 trials for datasets A, B, C, and D. Moreover, it can be seen from the data in Table 7 that all the computed average least lost values are exceedingly small in the range of 0.000–0.003 although the feature dimension was reduced to a fewer number of features, e.g., for dataset A the feature dimension were reduced from 2400 (in the original raw vibration signals) to 240 (the dimension of the compressively sampled signals with *α =* 0.1), which reduced to 13 (*d-dimension*) and then further reduced to 6 (*f-dimension with 0.01 tolerance value*).

**Figure 9:**
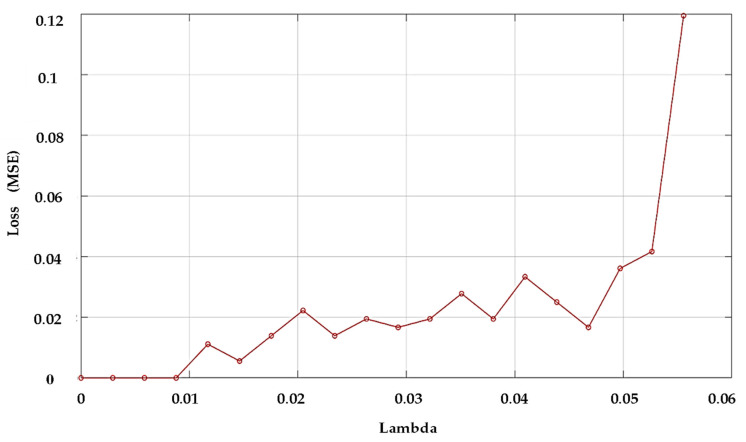
Example of the average loss values versus λ values computed from the reduced dimension of compressively sampled data with *α* = 0.2 and dataset A.

**Figure 10:**
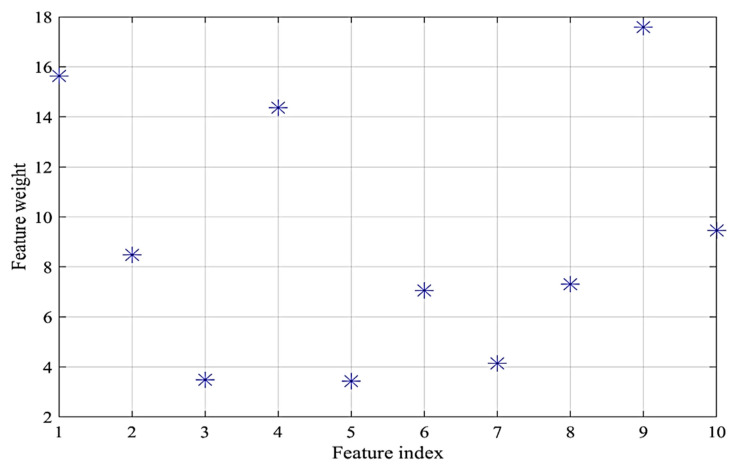
Example of the selected features from dataset A and their corresponding weights using *α* = 0.2 and NCA tolerance value = 0.02.

Moreover, the computed average best lambda values for the NCA algorithm are in the range of 0.0002–0.0041. The NCA tolerance values have a clear effect on the computed intrinsic dimension (*d*) and the dimension of the NCA-based selected features. For example, for dataset A with a 0.02 tolerance value, we obtained an intrinsic dimension (*d*) of 18, 21, and 25, which reduced to 13, 15, and 15 with 0.01 tolerance values for α=0.1, α=0.2, and α=0.3, respectively. Furthermore, for dataset A with the tolerance value of 0.02, we obtained the final feature dimension (*f*) of 10, 12, and 14, which reduced to 6, 7, 9 with 0.01 tolerance for α=0.1, α=0.2, and α=0.3, respectively. It can therefore be assumed that when the tolerance value decreases the dimension of both selected features, i.e., *d* and *f*, decreases. Figure 9 shows an example of the average loss values versus λ values. Figure 10 presents an example of the selected features and their corresponding weights.

The final NCA-based selected features from datasets A, B, C, and D were used to train the MLR classifier to obtain a trained classification model for each dataset to classify among *c* classes of roller bearing health conditions. Table 8 shows an overview of the testing results for each bearing vibration dataset where the classification accuracy is the average of 10 trials for each experiment, and the time obtained by averaging the testing time of these 10 trials. One of the more significant findings to emerge from the results in Table 8 is that the classification results with α=0.2 and 0.3 and tolerance values = 0.01 and 0.02 for all datasets A, B, C, and D are all over 99%. For datasets B and C with α=0.3 and tolerance values of 0.01 and 0.02, our proposed method attained 100% classification accuracy.

Similarly, for dataset A with α=0.3 and tolerance value of 0.02, our proposed method achieved 100% classification accuracy for every single run in our experiments. Furthermore, the trained classification models of our proposed method for all bearing vibration datasets A, B, C, D require less than 0.01 s to complete the classification task. These findings suggest that our proposed method is fast and offers high classification accuracies for rolling bearings from vibration datasets under different load levels as in A, B, and C.

Table 9 presents sample confusion matrices of the classification results of MLR classifier using selected features with tolerance value = 0.01 and a sampling rate of (a) α=0.1, 0.2, and 0.3 with dataset A. As can be seen from Table 9a with 10% testing data our method misclassified three of the testing examples of condition 6, i.e., IR2, as condition 1, i.e., NO, condition 3, i.e., RE2, and condition nine, i.e., OR2, respectively. In Table 9b, with 20% testing data, our method misclassified three of the testing examples of condition 6, i.e., IR2, as condition 7, i.e., IR3. Moreover, in Table 9c the recognition of all bearing health conditions is 100%.

#### Comparisons of Results

For additional assessment of the efficiency of the proposed method, Table 10 shows comparisons with some recently published results using the same bearing vibration datasets used in the second case study. In [4], a CS-DNN technique that involves a deep neural network method (DNN) with two hidden layers method combined with the Haar Wavelet-based CS technique was used to classify rolling bearing from the same rolling bearing datasets A, B, and C with α=0.1. In [38], a framework combining CS and feature ranking techniques including Fisher score, Laplacian score, Relief-F, Pearson correlation coefficients, and Chi-square (Chi-2) were used for bearing fault classification from the dataset D with α=0.1 and a feature dimension of 120. With these features, the MLR classifier was used to classify bearing faults. In [48], several methods were used to classify bearing faults with the same roller bearing the dataset D used in the second study. One of the methods used feature selection by adjunct rand index and standard deviation ratio (FSAR) the original feature set (OFS). Some of the other techniques utilized PCA, LDA, LFDA, and support margin LFDA (SM-LFDA). The selected features of these methods were used to train SVM to be used for bearing fault classification. Moreover, classification results for bearing fault classification using two methods with datasets A, B, and C are reported in [49]. The first method is based on a deep neural network (DNN) and the second method is a method based on a backpropagation neural network (BPNN). Additionally, classification results using a generic multi-layer perceptron (MLP) method with datasets A, B, and C are reported in [50].

As shown in Table 10, the results from our proposed method are better than those reported in [38,48,50]. Additionally, our classification results are better than the results produced using BPNN in [49]. Moreover, our results are the same as, if not better than the results reported in [4] and the results obtained using DNN in [49]. Remarkably, the results show that our method is faster than the CS-DNN technique that used in [4] as our method requires less than 0.005 s while the CS-DNN requires at least 5.7 s to complete the classification task.

In summary, the high reduction in the computation time originates from two reasons—(i) using CS that allow us to use a smaller sampling rate as in α=0.1, 0.2, and 0.3, and (ii) selecting far fewer features to be used for training the classification algorithm and for classifying rolling bearing health conditions using the trained classification model. Finally, our proposed method achieves classification results for all the rolling bearing vibration datasets A, B, C, and D that are the same as, if not more improved than, fault classification results from the literature on the same vibration bearing datasets.

## 4. Conclusions

The purpose of the present research was to examine the classification of bearing health conditions with far fewer selected features of compressively sampled vibration signals to achieve highly reduced computation time and yet to achieve high classification accuracy. The proposed method comprises a CS-based technique, which was used to obtain compressed vibration signals, followed by an intrinsic dimension estimation-based feature selection process that includes SPE-based feature selection with a self-organizing iterative scheme to embed the compressed data dimension into a further lower dimension, and a non-parametric NCA-based feature selection that maximize the stochastic variant of the leave-one-out nearest neighbour score to achieve the best classification accuracy on the training set. This ensures selecting fewer features from the high dimensional data capable of achieving high classification accuracy and a reduced computation time.

The multinomial logistic regression (MLR) algorithm was used to classify bearing faults. Two fault classification cases of rolling bearings vibration signals under different working loads were used to test the proposed method. The first set of analyses inspected the impact of the compressive sampling rate and the tolerance values on the number of the selected features The experimental results of bearing faults classification demonstrated that the proposed method could obtain higher classification accuracy and higher reduction in the computational time. The higher reduction in the computation time originates from two causes, (i) using CS that allows us to use a smaller sampling rate as in α=0.1, 0.2, and 0.3, and (ii) selecting far fewer features to be used for training the classification algorithm and for classifying rolling bearing health conditions using the trained classification model. Finally, our proposed method achieves classification results for all the rolling bearing vibration datasets A, B, C, and D that are as good as, if not better than, classification results from the literature on the same vibration bearing datasets.

## Figures and Tables

**Table 1:** The characteristics of bearings’ health conditions in the obtained bearing dataset.

Condition	Characteristic
NO	The bearing was brand new and in perfect condition.
NW	The bearing was in service for some time but in good condition.
IR	Inner race fault. This fault was created by cutting a small groove in the raceway of the inner race.
OR	Outer race fault. This fault was created by cutting a small groove in the raceway of the outer race.
RE	Roller element fault. This fault was created by using an electrical etcher to mark the surface of the balls, simulating corrosion.
CA	Cage fault. This fault was created by removing the plastic cage from one of the bearings, cutting away a section of the cage so that two of the balls were not held in a regular space and had freedom to move.

**Table 2:** Examples of the computed values of the average intrinsic dimension, the dimension of the NCA-based selected features, least loss, and best λ values taken from 10 trials.

NCA Tolerance Value	CS Sampling Rate (α)	Average Intrinsic Dimension (*d*)	The Average Dimension of NCA-Based Selected Features (*f*)	Average Least Loss	Average Best Lambda for NCA
0.01	0.1	28	8	0.013	0.004
	0.2	40	10	0.009	0.004
	0.3	26	8	0.010	0.003
0.02	0.1	62	18	0.014	0.003
	0.2	55	14	0.013	0.003
	0.3	33	11	0.013	0.003

**Table 3:** Classification results with their corresponding RMSE and computational time for the automatically selected features (*d* refers to the intrinsic dimension, and *f* is the dimension of the NCA-based selected feature) using two values of NCA tolerance and three compressive sampling rates.

NCA Tolerance Value	CS Sampling Rate (α)	*d*	*f*	MLR Classifier			
				Training Accuracy (%)	Training Time (s)	Testing Accuracy (%)	Testing Time (s)
0.01	0.1	28	8	99.8 ± 0.3	5.34 ± 1.7	99.5 ± 0.6	0.015 ± 0.002
	0.2	40	10	99.9 ± 0.1	4.6 ± 2.3	99.7 ± 0.3	0.003 ± 0.00
	0.3	26	8	100 ± 0.0	3.3 ± 0.5	99.9 ± 0.1	0.003 ± 0.001
0.02	0.1	62	18	99.9 ± 0.2	3.37 ± 0.8	99.7 ± 0.3	0.003 ± 0.001
	0.2	55	14	99.9 ± 0.1	3.55 ± 0.9	99.8 ± 0.2	0.003 ± 0.001
	0.3	33	11	100 ± 0.0	4.6 ± 2.0	100 ± 0.0	0.004 ± 0.003

**Table 4:** Sample confusion matrices of the classification results of MLR classifier using selected features with tolerance value = 0.01 and a sampling rate of (a) α=0.1, (b) α=0.2, and (c) α=0.3.

	NO	NW	IR	OR	RE	CA
**NO**	80	0	0	0	0	0
**NW**	0	80	0	0	0	0
**IR**	0	0	80	0	0	0
**OR**	0	0	0	80	0	0
**RE**	0	0	1	0	79	0
**CA**	0	0	0	0	0	80
(a)						
	**NO**	**NW**	**IR**	**OR**	**RE**	**CA**
**NO**	80	0	0	0	0	0
**NW**	0	78	0	0	0	2
**IR**	0	0	80	0	0	0
**OR**	0	0	0	80	0	0
**RE**	0	0	0	0	80	0
**CA**	0	0	0	0	0	80
(b)						
	**NO**	**NW**	**IR**	**OR**	**RE**	**CA**
**NO**	80	0	0	0	0	0
**NW**	0	80	0	0	0	0
**IR**	0	0	80	0	0	0
**OR**	0	0	0	80	0	0
**RE**	0	0	0	0	80	0
**CA**	0	0	0	0	0	80
(c)						

**Table 5:** A comparison with the classification results from the literature on the vibration bearing dataset of the first case study.

Ref	Method	Testing Accuracy	Testing Time
[33]	Raw vibration with entropic features + SVM	98.9 ± 1.2	_
	Compressed sampled with *α* = 0.5 followed by signal reconstruction + SVM	92.4 ± 0.5	
	Compressed sampled with *α* = 0.25 followed by signal reconstruction + SVM	84.6 ± 0.41	
[44]	GP generated feature sets (un-normalised data)		
	ANN	96.5	
	SVM	97.1	
[45]	FMM-RF SamEn	99.7 ± 0.02	_
	PS	99.7 ± 0.50	
	SamEn + PS	99.8 ± 0.41	
[36]	CPDC (with 6000 inputs from FFT)	99.4 ± 0.5	64.9
	CS-CPDC *α* = 0.1	99.8 ± 0.2	6.7
	α = 0.2	99.9 ± 0.1	7.8
[37]	With FFT, *α* = 0.1, feature dimension = 120, and LRC classifier)		_
	CS-FS	99.7 ± 0.4	
	CS-LS	99.5 ± 0.3	
	CS-Relief-F	99.8 ± 0.2	
	CS-PCC	99.8 ± 0.3	
	CS-Chi-2	99.5 ± 0.5	
[46]	Feature selection (with *λ* = 0.004, tolerance value = 0.02) from compressively sampled data and SVM for fault classification:		_
	α = 0.1 and feature dimension = 14	98.8 ± 2.4	
	α = 0.2 and feature dimension = 13	99.9 ± 0.2	
	α = 0.3 and feature dimension = 26	99.9 ± 0.1	
	Our proposed method with *λ* = 0.003, NCA tolerance value = 0.01, *α* = 0.1, and feature dimension = 8:		
	MLR classifier	99.5 ± 0.6	0.015
	SVM classifier	99.5 ± 0.5	0.060
	Our proposed method with *λ* = 0.003, NCA tolerance value = 0.01, *α* = 0.2, and feature dimension = 10:		
	MLR classifier	99.7 ± 0.3	0.003
	SVM classifier	99.8 ± 0.2	0.040
	Our proposed method with *λ* = 0.003, NCA tolerance value = 0.01, *α* = 0.3, feature dimension = 8:		
	MLR classifier	100 ± 0.0	0.003
	SVM classifier	100 ± 0.0	0.030

**Table 6:** Description of the bearing health conditions of the bearing vibration dataset used in the second case study.

Health Condition	Fault Width (mm)	Classification Label
NO	0	1
RE1	0.18	2
RE2	0.36	3
RE3	0.53	4
RE4	0.71	5
IR1	0.18	6
IR2	0.36	7
IR3	0.53	8
IR4	0.71	9
OR1	0.18	10
OR2	0.36	11
OR3	0.53	12

**Table 7:** Examples of the computed values of the average intrinsic dimension, the dimension of the NCA-based selected features, least loss, and best λ values taken from 10 trials for datasets A, B, C, and D.

Dataset	NCA Tolerance Value	CS Sampling Rate (α)	*d*	*f*	Average Least Loss	Average Best Lambda for NCA
A	0.01	0.1	13	6	0.001	0.0011
		0.2	15	7	0.000	0.0009
		0.3	15	9	0.001	0.0006
	0.02	0.1	18	10	0.000	0.0009
		0.2	21	12	0.000	0.0004
		0.3	25	14	0.000	0.0003
B	0.01	0.1	17	7	0.000	0.0007
		0.2	19	9	0.001	0.0006
		0.3	24	9	0.000	0.0002
	0.02	0.1	28	11	0.000	0.0009
		0.2	23	10	0.000	0.0005
		0.3	26	12	0.000	0.0003
C	0.01	0.1	16	5	0.001	0.0010
		0.2	17	7	0.000	0.0006
		0.3	23	8	0.000	0.0006
	0.02	0.1	21	11	0.000	0.0009
		0.2	22	12	0.000	0.0004
		0.3	27	14	0.000	0.0003
D	0.01	0.1	15	4	0.003	0.0041
		0.2	18	5	0.001	0.0041
		0.3	20	7	0.001	0.0015
	0.02	0.1	17	9	0.001	0.003
		0.2	21	10	0.001	0.003
		0.3	23	9	0.001	0.003

**Table 8:** Classification results with their corresponding RMSE and computational time for the automatically selected features (*d* refers to the intrinsic dimension, and *f* is the dimension of the NCA-based selected feature) using two values of the NCA tolerance and compressive sampling rates for datasets A, B, C, and D (all classification accuracies of 100% are in bold).

Dataset	NCA Tolerance Value	CS Sampling Rate (α)	*d*	*f*	MLR Classifier	
					Testing Accuracy (%)	Testing Time (s)
A	0.01	0.1	13	6	98.5 ± 0.7	0.002
		0.2	15	7	99.9 ± 0.1	0.003
		0.3	15	9	99.9 ± 0.1	0.002
	0.02	0.1	18	10	99.6 ± 0.2	0.003
		0.2	21	12	99.8 ± 0.2	0.006
		0.3	25	14	**100 ± 0.0**	0.003
B	0.01	0.1	17	7	99.2 ± 0.7	0.002
		0.2	19	9	99.5 ± 0.5	0.003
		0.3	24	9	**100 ± 0.0**	0.009
	0.02	0.1	28	11	99.9 ± 0.1	0.003
		0.2	23	10	99.9 ± 0.1	0.006
		0.3	26	12	**100 ± 0.0**	0.003
C	0.01	0.1	16	5	99.7 ± 0.2	0.002
		0.2	17	7	99.9 ± 0.1	0.003
		0.3	23	8	**100 ± 0.0**	0.002
	0.02	0.1	22	11	99.9 ± 0.1	0.003
		0.2	27	12	99.9 ± 0.1	0.006
		0.3	15	14	**100 ± 0.0**	0.003
D	0.01	0.1	18	4	92.7 ± 2.9	0.002
		0.2	20	5	99.1 ± 0.8	0.002
		0.3	17	7	99.9 ± 0.1	0.002
	0.02	0.1	21	9	99.9 ± 0.1	0.002
		0.2	23	10	99.9 ± 0.1	0.002
		0.3	13	9	99.9 ± 0.1	0.002

**Table 9:** Sample confusion matrices of the classification results of MLR classifier using selected features with tolerance value = 0.01 and a sampling rate of (a) α=0.1, (b) α=0.2, and (c) α=0.3  with the dataset A.

	**NO**	**RE1**	**RE2**	**RE3**	**IR1**	**IR2**	**IR3**	**OR1**	**OR2**	**OR3**
**NO**	100	0	0	0	0	0	0	0	0	0
**RE1**	0	100	0	0	0	0	0	0	0	0
**RE2**	0	0	100	0	0	0	0	0	0	0
**RE3**	0	0	0	100	0	0	0	0	0	0
**IR1**	0	0	0	0	100	0	0	0	0	0
**IR2**	1	0	1	0	0	97	0	0	1	0
**IR3**	0	0	0	0	0	0	100	0	0	0
**OR1**	0	0	0	0	0	0	0	100	0	0
**OR2**	0	0	0	0	0	0	0	0	100	0
**OR3**	0	0	0	0	0	0	1	0	0	99
(a)										
	**NO**	**RE1**	**RE2**	**RE3**	**IR1**	**IR2**	**IR3**	**OR1**	**OR2**	**OR3**
**NO**	100	0	0	0	0	0	0	0	0	0
**RE1**	0	100	0	0	0	0	0	0	0	0
**RE2**	0	0	100	0	0	0	0	0	0	0
**RE3**	0	0	0	100	0	0	0	0	0	0
**IR1**	0	0	0	0	100	0	0	0	0	0
**IR2**	0	0	0	0	0	97	3	0	0	0
**IR3**	0	0	0	0	0	0	100	0	0	0
**OR1**	0	0	0	0	0	0	0	100	0	0
**OR2**	0	0	0	0	0	0	0	0	100	0
**OR3**	0	0	0	0	0	0	0	0	0	100
(b)										
	**NO**	**RE1**	**RE2**	**RE3**	**IR1**	**IR2**	**IR3**	**OR1**	**OR2**	**OR3**
**NO**	100	0	0	0	0	0	0	0	0	0
**RE1**	0	100	0	0	0	0	0	0	0	0
**RE2**	0	0	100	0	0	0	0	0	0	0
**RE3**	0	0	0	100	0	0	0	0	0	0
**IR1**	0	0	0	0	100	0	0	0	0	0
**IR2**	0	0	0	0	0	100	0	0	0	0
**IR3**	0	0	0	0	0	0	100	0	0	0
**OR1**	0	0	0	0	0	0	0	100	0	0
**OR2**	0	0	0	0	0	0	0	0	100	0
**OR3**	0	0	0	0	0	0	0	0	0	100
(c)										

**Table 10:** A comparison with the classification results from the literature on the vibration bearing datasets A, B, C, and D of the second case study.

Ref	Dataset	Method	Testing Accuracy (%)	Testing Time (s)
	A		99.3 ± 0.6	5.7
[4]	B	CS-DNN with *α* = 0.1	99.7 ± 0.5	5.9
	C		100 ± 0.0	5.7
[37]	D	With FFT, *α* = 0.1, feature dimension = 120, and LRC		_
		CS-FS	98.4 ± 1.6	
		CS-LS	99.1 ± 0.8	
		CS-Relief-F	99.3 ± 0.6	
		CS-PCC	99.2 ± 0.8	
		CS-Chi-2	97.5 ± 2.6	
[47]		OFS-FSAR-SVM		_
		Selected features = 25	91.46	
		Selected features = 50	69.58	
		OFS-FSAR-PCA-SVM		
		Selected features = 25	91.67	
		Selected features = 50	69.79	
		OFS-FSAR-LDA-SVM		
		Selected features = 25	86.25	
		Selected features = 50	92.70	
		OFS-FSAR-LFDA-SVM		
		Selected features = 25	93.75	
		Selected features = 50	94.38	
		OFS-FSAR-(SM-LFDA)-SVM		
		Selected features = 25	94.58	
	D	Selected features = 50	95.63	
[48]	A		99.95 ± 0.06	_
	B		99.61 ± 0.21	
	C	DNN	99.74 ± 0.16	
	A		62.20 ± 18.09	
	B		61.95 ± 22.09	
	C	BPNN	69.82 ± 17.67	
[49]	A B C		MLP	_
		Our proposed method with *λ* = 0.003, NCA tolerance value = 0.02, and *α* = 0.1.		
	A	feature dimension = 10	99.6 ± 0.2	0.003
The proposed	B	feature dimension = 11	99.9 ± 0.1	0.003
method	C	feature dimension = 11	99.9 ± 0.1	0.003
	D	feature dimension = 9	99.9 ± 0.1	0.002

## Data Availability

The data presented in the first case study may be available on request from the first author, Hosameldin O. A. Ahmed.
